# Perturbation walking effects on prefrontal cortical activation and walking performance in older women with and without osteoarthritis: a FNIRS study

**DOI:** 10.3389/fnagi.2024.1403185

**Published:** 2024-08-22

**Authors:** Alka Bishnoi, Yang Hu, Manuel E. Hernandez

**Affiliations:** ^1^Department of Physical Therapy, College of Health Professions and Human Services, Kean University, Union, NJ, United States; ^2^Department of Kinesiology, San Jose State University, San Jose, CA, United States; ^3^Department of Biomedical and Translational Sciences, Carle Illinois College of Medicine, University of Illinois Urbana-Champaign, Urbana, IL, United States; ^4^Department of Kinesiology and Community Health, College of Applied Health Sciences, University of Illinois Urbana-Champaign, Urbana, IL, United States

**Keywords:** prefrontal cortex, gait, functional near infrared spectroscopy, walking, osteoarthiritis

## Abstract

**Introduction:**

Perturbation walking (PW) has been shown to improve gait, however its effect on the cortical control of gait might provide insights on neural mechanisms underlying falls in adults with osteoarthritis. The objective of this study is to investigate the effect of PW on prefrontal cortical (PFC) activation in older women with (OA) and without osteoarthritis (HOA). We hypothesized that there would be an increase in PFC activation during PW relative to comfortable walking (CW) and higher increase in PFC activation during PW in HOA compared to OA.

**Methods:**

Twenty community-dwelling older women (66.7 ± 5.41 years old) walked on an instrumented treadmill that provided perturbations at pseudo-random intervals between 5–25 s using a counterbalanced design. Functional Near Infrared Spectroscopy was used to quantify PFC oxygenated hemoglobin (HbO_2_) and deoxyhemoglobin (Hb) levels, while standing prior to the task as a baseline. A linear mixed effects model was conducted to investigate the effects of cohort (HOA vs OA), task (PW vs CW), and their interaction on HbO_2_ (μM) and Hb (μM) levels.

**Results:**

HbO_2_ and Hb levels differed significantly between CW and PW tasks for both cohorts (*P* < 0.001) and demonstrated significant task by cohort interaction (*P* < 0.05). In addition, we found changes in walking performance (stride time, stride length, stride width and stance time) during and after PW. Spearman correlation demonstrated a strong association between increased stance time, increased body mass index and decreased PFC activation during PW. No other significant results were found.

**Discussion:**

This study found increase in PFC activation during PW and gait adaptation after a short bout of PW in HOA and OA. This increase in PFC activation was higher in HOA compared to OA, particularly during PW tasks, and was consistent with theory of limitations in mobility affecting neural activation in older adults. Further work remains to examine how pain, obesity, and mobility impacts cortical control in older adults with and without osteoarthritis.

## 1 Introduction

Osteoarthritis is a common joint disease in the United States (CDC, 2020), with an increasing prevalence in older adults, particularly women over 50 years of age (Shane Anderson and Loeser, 2010). In addition to age and gender, other major risk factors for osteoarthritis include joint injury or overuse, obesity, and genetic factors that contribute to decreased ability for joint tissues to compensate for abnormal mechanical stresses (Shane Anderson and Loeser, 2010). Due to joint pain, stiffness, and inflammation, older adults with osteoarthritis have decreased range of motion leading to increase in mobility impairment, decline in physical activity and increase in hospitalization (Shane Anderson and Loeser, 2010; Ng and Tan, 2013; Wollesen and Voelcker-Rehage, 2019). In addition to these symptoms, osteoarthritis is associated with depression, muscle weakness and instability (Ng and Tan, 2013), leading to mobility impairment in older adults (Guilak, 2011). This mobility impairment alters the walking pattern of the individual, putting additional stress and strain on joints (Messier, 1994; Guilak, 2011). Decrease in mobility impairment can be achieved by increasing physical activity in older adults with osteoarthritis through intervention strategies (Bhatt et al., 2012, 2018).

Intervention strategies such as perturbation walking (PW) have been used traditionally to recover balance in adults with osteoarthritis (Fitzgerald et al., 2011; Krasilshchikov et al., 2018; Rutherford et al., 2022). PW exposes the participant to quick starts and stops, or changes in direction while walking (Bhatt et al., 2012; Pai et al., 2014). Previous studies have examined the effects of PW in adults with osteoarthritis to challenge balance (Fitzgerald et al., 2011), which can improve physical function and decrease risk of falls (Fitzgerald et al., 2011; Pai et al., 2014; Krasilshchikov et al., 2018; Rutherford et al., 2022). Benefits of PW include reduction in self-reported pain, improvement in self-reported function (Fitzgerald et al., 2011), decreased falls (Pai et al., 2014; Krasilshchikov et al., 2018), and improvement in muscle activation (Rutherford et al., 2022). PW has also been used in older adults to improve balance and decrease fall risk by improving stepping execution and dynamic stability (Bhatt et al., 2012; Kurz et al., 2016). PW has repeatedly shown to be an effective tool in improving dynamic gait and balance (Bhatt et al., 2005, 2012; Pai et al., 2014; Kurz et al., 2016; Krasilshchikov et al., 2018; Rutherford et al., 2022), however, its effect on cortical activation and walking function in adults with osteoarthritis hasn’t been explored yet.

Decline in dynamic gait and balance has been shown to be associated with decline in executive functioning due to the loss of gray matter and changes in white matter connectivity in healthy older adults (Demnitz et al., 2017). However, given the decline in physical functioning and neurological changes in older adults with osteoarthritis due to pain and inflammation, an open question remains if older adult with osteoarthritis would be able to modulate cortical activation similar to healthy older adults during PW. Given observations of a significant deactivation of prefrontal cortical (PFC) activity with increased pain in older adults with osteoarthritis (Oztürk et al., 2021) and decreases in dorsolateral PFC activity with increased pain sensitivity (Lev et al., 2013), increased chronic pain expected in older women with osteoarthritis would be expected to decrease the availability of cortical resources in PFC for recruitment during PW.

The use of functional near infrared spectroscopy (fNIRS) while walking has been demonstrated to provide reliable and valid measures of PFC activity in older adults, as noted by a recent review (Bishnoi et al., 2021). Furthermore, in PW tasks, cortical changes in control have been confirmed using either electroencephalography (EEG) (Peterson and Ferris, 2018) or fNIRS (Koren et al., 2019; Lee et al., 2023), consistent with the use of increased attentional resources during PW. However, the examination of changes in the use of attentional resources in older adults due to unexpected perturbations while walking, which provide an analogue to walking on icy surfaces in the community, merit further investigation.

The objective of this cross-sectional study was to investigate the effects of PW on PFC activation using fNIRS and walking performance in older women with and without osteoarthritis. We hypothesized that there would be an increase in PFC activation in older women during PW, relative to comfortable walking (CW) and this increase would be greater in healthy controls compared to older women with osteoarthritis, due to reductions in the availability of neural resources due to chronic pain (Seidler et al., 2010). In addition to this, we expected an improvement in walking performance after PW tasks based on previous studies including on PW tasks (Madehkhaksar et al., 2018). This study will further our understanding of brain activation changes explored in older women with osteoarthritis during challenging walking tasks. Future studies can evaluate the benefits of PW as an intervention to improve the overall functional capacity of older adults with osteoarthritis.

## 2 Materials and methods

### 2.1 Participants

Twenty community-dwelling older females (66.7 ± 5.41 years of age [Mean ± SD]) were recruited for this two-session cross-sectional laboratory study and divided into two groups, healthy older adults (HOA, *n* = 11) and older adults with osteoarthritis (OA, *n* = 9) based on their reported diagnosis. Inclusion criteria consisted of no report of any neurological condition, cardiovascular condition, no physical disabilities, and able to walk independently without any assistive device. Exclusion criteria consisted of score less than 19 on the Telephone Interview for Cognitive Status (Welsh et al., 1993) or inability to walk without assistive device. All participants signed a written consent form approved by the local institutional review board. Based on prior work examining the differences in hemodynamics during painful stimuli in older adults with osteoarthritis (Oztürk et al., 2021), a sample size of 8 OA and 8 HOA participants was needed to detect a significant effect at the *p* = 0.05 level with.90 power and an effect size *f* = 0.8, based on an *a priori* sample size analysis using G*Power (Version 3.1.9.6).

### 2.2 Protocol

All participants made two separate visits to the mobility and fall prevention laboratory situated at the University of Illinois, Urbana-Champaign. On day 1, participants provided informed consent, performed physical and cognitive assessments, and received treadmill training. On Day 2, participants performed CW and PW tasks at a fixed comfortable walking speed.

### 2.3 Physical and cognitive assessments

Participants had their cognitive function assessed using the Repeated Battery for the Assessment of Neuropsychological Status (RBANS) (Randolph et al., 1998), and Trail Making Test (TMT) (Ashendorf et al., 2008). Education was assessed as the number of years of higher education post high school. During physical assessment, participants performed several motor behavioral tests including the Mini Balance Evaluation Systems Test (Mini-BEST) (Yingyongyudha et al., 2016), Short Physical Performance Battery Test (SPPB) (Veronese et al., 2014), and single and dual task Timed Up and Go (TUG). The depression was assessed using geriatric depression scale (GDS) (Kurlowicz and Greenberg, 2007). Pain was assessed using self-reported Western Ontario and McMaster Universities Osteoarthritis Index assessing activity and pain levels (WOMAC) subscale (Salaffi et al., 2003).

### 2.4 Perturbation walking task

After baseline testing on day 1, a researcher determined the comfortable speed of the participant by starting the treadmill speed at 0.7 m/s and increased the speed until the participant felt that the speed was not too fast or too slow. After finding the comfortable walking (CW) speed, participants did a training session of CW fixed speed for 5 min on the instrumented treadmill (C-Mill, Motekforce link, Culemborg, The Netherlands). On day 2, participants performed two CW fixed speed tasks of 2 min each and two PW tasks on an instrumented treadmill (C-Mill, Motekforcelink, Culemborg, The Netherlands), where the treadmill belt provided anterior-posterior perturbations at pseudo random intervals between 5–25 s using a counterbalanced design. During each PW task, there were 10 perturbations during the 120 s interval, averaging it to 1 perturbation every 20 s. In PW tasks, custom belt speed profiles were used to simulate slips at pseudorandom intervals between 5 and 25 s, which subjects were asked to recover from as best as possible while they were walking at a comfortable pace. In specific, a perturbation profile with a brief deceleration of up to −5 m/s^2^ for up to 0.2 s, followed by an acceleration of up to 5 m/s^2^ for up to 0.2 s was employed to examine the participant’s recovery response to a partial slip. The tasks took place in the following order: first comfortable walking task (CW1), first perturbation walking task (PW1), second perturbation walking task (PW2), and second comfortable walking task (CW2). Participants completed the PW on the treadmill while wearing a harness and an fNIRS headband. Their instruction for PW tasks was to recover as best as possible to each perturbation.

### 2.5 Spatiotemporal gait data

Spatiotemporal data and gait event data using online gait event detection (Roerdink et al., 2008) were collected during gait assessments using CueFors 2 software (Motekforce Link, Culemborg, The Netherlands), which is equipped in the C-Mill instrumented treadmill. Spatiotemporal gait characteristics, including stride time (StrT) (seconds), stride length (StrL) (m), stride width (StrW) (m), and stance time (StaT) (seconds) were calculated using custom python scripts, as described and defined in prior work (Kaur et al., 2021). Stride time is the time between two successive heel strikes (i.e., right heel strike to right heel strike). Stride length is the anteroposterior distance between two subsequent heel strikes of the same foot, while adjusting for belt travel. Stride width is the medio-lateral distance between the two feet (i.e. perpendicular distance between the line connecting two consecutive heel strikes of the same foot). Stance time is the time between heel strike and toe off of the same foot. Throughout each walking trial, ground reaction forces, treadmill speed, and center of pressure position coordinates were recorded at 500 Hz while position and time of gait events were calculated using CueFors 2 software.

### 2.6 Functional near infrared spectroscopy

FNIRS data was obtained using an fNIRS Imager 1200 system (fNIRS Devices, LLC, Potomac, MD). The headband sensor contained 10 photodetectors and 4 LED light sources with a 2.5 cm source detector separation distance that covered the forehead with the use of 16 optodes and a 2Hz sampling rate. The light sources on the sensor (Epitex Inc. type L6 × 730/6 × 850) contain two built-in LEDs having peak wavelengths at 730 and 850 nm, with an overall outer diameter of 9.2 ± 0.2 mm. The photodetectors (Bur Brown, type OPT101) are monolithic photodiodes with a single supply transimpedance amplifier. The center of the headband sensor was placed on the central point of the forehead above the nasion (Fpz) in accordance with the 10/20 electroencephalography system. Relative HbO_2_ and Hb levels (μM) were used because of their reliability in evaluating cortical activation changes (Ferrari and Quaresima, 2012). The fNIRS data were collected using COBI Studio software and processed and analyzed using custom MATLAB scripts. Visual inspection of raw data was used to monitor for excessive noise, saturation, or dark current conditions. To minimize the effects of physiological artifacts (e.g., breathing and heart rate) and any additional noise, the raw data were filtered using a low-pass filter with a cut-off frequency at 0.14Hz. HbO_2_ and Hb levels (μM) were then calculated using the modified Beer-Lambert law for each of the 16 channels (Hamacher et al., 2015).

PFC activation levels were assessed during the two CW and two PW tasks. The whole duration of the task was used to calculate the mean PFC activation. Each task started and ended with 10s of standing quietly. All participants were asked to look forward and count silently starting from 1 in their head. After that 10s baseline, the instructions for a specific task were then given. The 10s baseline before each task was used as a reference for both HbO_2_ and Hb relative levels (μM) (Ohsugi et al., 2013). The task-related changes were measured by averaging Hb and HbO_2_ levels during the walking tasks and comparing it to baseline value just prior to each condition. Individual mean HbO_2_ and Hb values were extracted separately for each of the 16 optodes in each task.

### 2.8 Statistical analysis

Descriptive statistics for age, body mass index (BMI), RBANS, comfortable gait speed, WOMAC, GDS, single and dual task TUG were reported in Table 1 with differences between cohorts evaluated using an independent t-test. Linear mixed effects model was used to evaluate any significant differences between HOA and OA, as a two-level between subject factor, walking task as a four-level repeated within-subject factor (CW1, PW1, CW2, PW2), and PFC activation, as measured by mean HbO_2_ levels and Hb levels as the dependent measure while controlling for repeated measures across the 16 optodes in analysis. The interaction term of cohort-by-task and random intercept was also included in the model to allow the entry point to vary across individuals. Data was visually inspected for normality and homogeneity of variance and the assumptions for the linear mixed effects model were met by all models. A linear mixed effects model was also used to examine the effect of group and task, and its interaction on spatiotemporal gait parameters (mean stride time, stance time, stride length and stride width). Lastly, a spearman correlation was used between dependent and independent variables. For all statistical tests, R version 3.1.1. was used, and significance was set at *p* < 0.05. Supplemental figures are provided to demonstrate group differences across PW1 and PW2 and correlation analyses.

**TABLE 1:** Participant characteristics (Mean (SD) presented).

Characteristics	HOA (*n* = 9)	OA (*n* = 11)	*P*-Value
Age (years)	67.7 ± 6.6	65.8 ± 3.2	0.200
BMI (kg/m^2^)	22.2 ± 3.1	27.1 ± 5.4	0.016*
RBANS [0–130]	111.7 ± 14.5	104.9 ± 9.2	0.110
TUG-ST (seconds)	9.7 ± 1.2	11.5 ± 1.2	0.003**
TUG-DT (seconds)	11.1 ± 1.9	13.1 ± 2.5	0.037*
WOMAC [0–20]	0.3 ± 0.5	3.4 ± 2.8	0.005**
CGS (m/s)	1.5 ± 0.3	1.3 ± 0.2	0.092
GDS [0–15]	0.4 ± 0.7	1.1 ± 1.4	0.092

CGS, Comfortable Gait Speed; BMI, body max index; TUG, Time up and Go test; ST, Single Task; DT, Dual Task; WOMAC, Western Ontario and McMaster Universities Osteoarthritis Index assessing activity and pain levels; RBANS, repeated battery for the for the assessment of neurophysiological status; GDS, Geriatric Depression Scale. Table is reported as Mean (SD).

Significance set at *p < 0.05,

**p < 0.01.

## 3 Results

### 3.1 Participants

After doing independent sample t-test for group differences, we found that there was a significant difference between HOA and OA in single (*p* = 0.003) and dual timed up and go test (*p* = 0.037), WOMAC (*p* = 0.005) and BMI (*p* = 0.016). No other significant differences were observed between groups for age (*p* = 0.200), RBANS (*p* = 0.110), gait speed (*p* = 0.092), and GDS score (*p* = 0.092) (Table 1).

### 3.2 PFC activation

**FIGURE 1:**
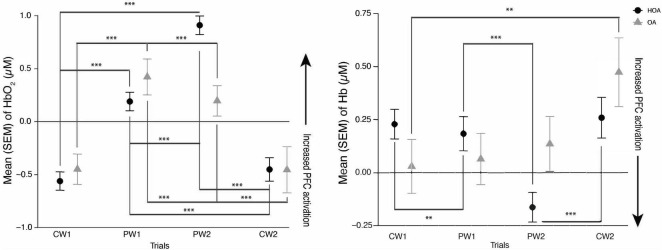
(Left) mean hemoglobin (Hb02) and (Right) mean deoxyhemoglobin (Hb) activation during comfortable walking (CW1, CW2) and perturbation walking tasks (PW1, PW2) in healthy older adults (HOA) and older adults with osteoarthritis (QA). ***p < 0.001, **p < 0.01.

A linear mixed effects model showed a significant increase in PFC activation levels during the PW tasks relative to CW tasks in both cohorts. During PW2, there was decrease in PFC activation in OA, compared to HOA (*p* < 0.001). There was also a significant interaction between cohort (OA) and PW2 task (*p* < 0.01), and cohort (OA) and CW1 task (*p* = 0.041) (Table 2). *Post hoc* Tukey’s analysis showed that there were PFC activation differences in between the tasks in both cohorts, but no overall changes between the cohorts were found (Figure 1).

**TABLE 2:** Results from linear mixed model for mean oxyhemoglobin (HbO_2_), deoxyhemoglobin (Hb), stride time (StrT), stride length (StrL), stance time (StaT) and stride width (StrW).

Effects	Estimate	SE	*p*-Value
**Mean HbO_2_ (μM)**			
Task: PW1	0.550	0.122	<0.001***
Task: PW2	1.37	0.117	<0.001***
Cohort OA: TaskPW2	−0.54	0.197	0.006**
**Mean Hb (μM)**			
Task: PW2	−0.32	0.096	0.0007***
Cohort OA: TaskCW1	0.317	0.155	0.041*
Cohort OA: TaskPW2	0.471	0.162	0.003**
**Mean StrT (s)**			
Task: PW1	0.03	0.009	< 0.01**
**Mean StrL (m)**			
Task: PW1	0.02	0.01	0.04*
**Mean StaT (s)**			
lTask: PW1	0.02	0.006	0.01*
**Mean StrW (m)**			
Task: PW2	0.008	0.003	<0.004**
Task: CW2	0.010	0.003	<0.0005***

Significance set at *p < 0.05,

**p < 0.01,

***p < 0.001; SE, Standard Error.

### 3.3 Gait parameters

**FIGURE 2:**
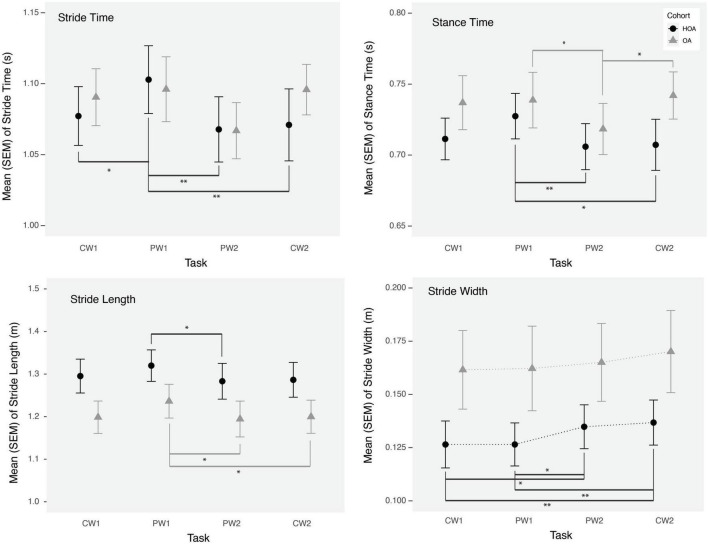
Mean stride time, stride length, stance time, and stride width during comfortable walking (CW1, CW2) and perturbation walking tasks (PW1, PW2) in healthy older adults (HOA) and older adults with osteoarthritis (OA). **p < 0.01, *p < 0.05.

While no walking speed comparisons were performed between PW and CW tasks due to the fixed belt speed used in tasks, we evaluated changes in spatiotemporal walking performance during and after PW using linear mixed model analysis. Stride time, stance time and stride length changed significantly from CW1 to PW1 task for both cohorts (Table 2). We found increases in stride time (*p* < 0.01), stance time (*p* = 0.01) and stride length (*p* = 0.04) during PW1, on the other hand, stride width changed significantly from CW1 to PW2 (*p* = 0.004) and from CW1 to CW2 (*p* < 0.0005) (Figure 2). Supplemental analysis was performed to quantify groups differences across PW1 and PW2 presented in supplementary file (Figure 1).

### 3.3 Correlation

Spearman correlations were used to check the association between dependent and independent variables (see Supplementary Appendix A). Through spearman correlation, we found a significant negative association between mean HbO_2_ and BMI (rho = −0.66, *p* = 0.003) during PW2 task for both cohorts. For spatiotemporal gait parameters association with PFC activation measures, we found a significant negative association between mean HbO_2_ and mean stance time (rho = −0.63, *p* = 0.008) during PW2 task for both cohorts.

## 4 Discussion

### 4.1 Primary findings of the study

This is the first study investigating the PFC activation and spatiotemporal gait differences between older adults with and without osteoarthritis while doing perturbation and comfortable walking tasks. Consistent with our hypothesis and prior work in adults (Koren et al., 2019), we found that both groups showed increased PFC activation during PW in comparison to CW. Furthermore, while similar increases in PFC activation were seen by both groups from CW1 to PW1 trials, we found that adults with OA didn’t demonstrate a comparable increase in PFC activation as evaluated by both Hb and HbO_2_ from CW1 to PW2 trials, as was seen in HOA. This might be due to a decrease in neural reserve related to mobility impairments or neurological changes in osteoarthritis as there was a decrease in PFC activation particularly in OA during their PW2 task. The findings in this study are consistent with a “supply and demand” framework in healthy adults (Seidler et al., 2010), as HOA might have demonstrated this increase in PFC activation during the more challenging PW tasks, relative to OA, due to an increased availability of cognitive resources. These findings are also consistent with the results of a recent study showing increases in prefrontal cortical activation as unevenness in terrain increases in older adults with different mobility function (Hwang et al., 2024).

In addition to PFC activation changes, we found significant effects on gait parameters, during the initial exposure to perturbations and following a short bout of PW in both groups. During the initial exposure of perturbations, both groups demonstrated an increase in stance time, stride time and stride length, which returned to baseline values by the next task. Furthermore, an increased stride width was observed after a short bout of PW during the PW2 task and afterward in both groups, which demonstrates a potentially adaptive strategy to reduce loss of balance or fall risk (Bhatt et al., 2013). Although, we didn’t find any association of stride width with PFC activation variables, we did find a negative association between PFC activation (mean HbO_2_) and stance time during the PW2 task, consistent with the use of increased attentional resources for restoring gait performance. Furthermore, similar to prior findings (Meester et al., 2014) in healthy young adults, we found that central mechanisms are activated in these adults in response to more demanding walking conditions. However, while reflex function and gait performance in young adults has demonstrated independence from cortical activation (Meester et al., 2014), increased neural activation may be needed for maintaining gait performance during PW conditions in these adults.

### 4.2 BMI and PFC activation

The findings of an association between neural activation during a challenging walking task and BMI differ from prior work (Osofundiya et al., 2016). While neural activation increases during walking have been observed in obese adults, particularly when precise gait is required (Osofundiya et al., 2016), differences in task, baseline selection, and co-morbidities may contribute to the differences observed in our study. In particular, decreased neural activation during the PW2 task was associated with higher BMIs in HOA and OA in this study, which may have been due to increased physical demands or central fatigue from the PW tasks on a treadmill with a fixed speed, in comparison to prior work in overground walking. Furthermore, while prior work used a single baseline at the start of the experiment, we used a unique baseline prior to the start of each task, for controlling hemodynamic changes during the experiment. Lastly, as increased BMI was often observed in OA in this study, further work disassociating the effect of body composition and musculoskeletal conditions is needed.

### 4.3 Motor performance

Previous perturbation studies have looked at healthy young adults, measuring gait measures and neural activation through fMRI before and after perturbation training (Bhatt et al., 2018), and found younger adults to take less compensatory steps after a week of perturbation training and higher levels of neural activation post-training compared to pre-training (Bhatt et al., 2018). We found a similar increase in neural activity and decrease of initial perturbation task changes after a short bout of PW. While no significant differences in gait performance were observed between HOA and OA, the lower increases in neural activation during the more challenging PW conditions in OA, may be arising from changes due to increased mobility impairment and pain, but further examination is needed in a cohort with a wider range of functional capacity.

### 4.4 Pain and cognitive function

Chronic pain has been linked to injurious falls in older adults (Leveille, 2009; Cai et al., 2021). In older adults with knee pain, it is found to be twice as likely to fall compared to healthy controls (Hicks et al., 2020). As osteoarthritis often leads to increased joint stiffness and inflammation, it can cause large amounts of pain during physical activity (Litwic et al., 2013). Furthermore, chronic pain has been shown to be associated with cognitive impairment (Apkarian et al., 2004; Gwilym et al., 2010; Parksl et al., 2011; Bell et al., 2022), due to affected cognitive flexibility leading to impairment in executive function while walking (Bell et al., 2022). While cohort differences in pain and mobility function were observed in this study, WOMAC pain scores were not found to be associated with PFC activation, which may be due to small sample size with low levels of pain in our study.

### 4.5 Clinical applications and future recommendations

Cortical activity measures provide an important lens on early changes in the control of walking in older adults (Braver et al., 1997; Verstynen et al., 2005). This study highlights changes in cortical control during the adaptation to more challenging walking conditions and may provide a complementary measure to evaluate the effectiveness of perturbation-based walking in older adults with osteoarthritis. PW can be a useful tool to aide those with osteoarthritis and improve cortical outcomes to control gait (Rutherford et al., 2022). Thus, by recognizing the differences in PFC activation in both groups, we can further refine targets for the rehabilitation to emphasize improvements in both gait and cortical control measures (Clark et al., 2021).

### 4.6 Limitations of the study

The present study has several key limitations. First, the sample size of the study was small, which limits generalizability. Secondly, we incorporated a two-day protocol only, longer periods of PW and an evaluation of retention of learned changes would be beneficial. Lastly, we examined only PFC activation, due to limited spatial coverage of the fNIRS device. Future studies are needed to examine the differences in other cortical areas.

## 5 Conclusion

In conclusion, this study found an increase in PFC activation during PW and gait adaptation after a short bout of PW in older women with and without osteoarthritis. This increase in PFC activation was higher in HOA compared to OA, particularly in PW tasks, is consistent with limitations in mobility affecting neural activation in older adults.

## Data Availability

The raw data supporting the conclusions of this article will be made available by the authors, without undue reservation.
