# Supplementary material for: Perturbation walking effects on prefrontal cortical activation and walking performance in older women with and without osteoarthritis: a FNIRS study
Source: Front Aging Neurosci. 2024 Aug 22;16:1403185. doi: 10.3389/fnagi.2024.1403185 (PMC11374618; doi:10.3389/fnagi.2024.1403185)
Supplement: Supplementary file 1 [file Data_Sheet_1.docx]

**Appendix A**

1. **Gait parameter changes over PW1 and PW2 trials:**

**Methods.** Customized Python scripts extracted stride time, stride length, stance time, and stride width from the center of pressure data and gait event data recorded by instrumented treadmill. Gait parameter (e.g., stride time, stride length, stance time, and stride width) errors were calculated by using the average value of five responsive strides after each perturbation (numbered A through J) and subtracting the local baseline calculated before each perturbation.

**Results.** Qualitatively, both older women with and without OA demonstrated similar decreases in gait parameter differences across PW1 and PW2 trials.


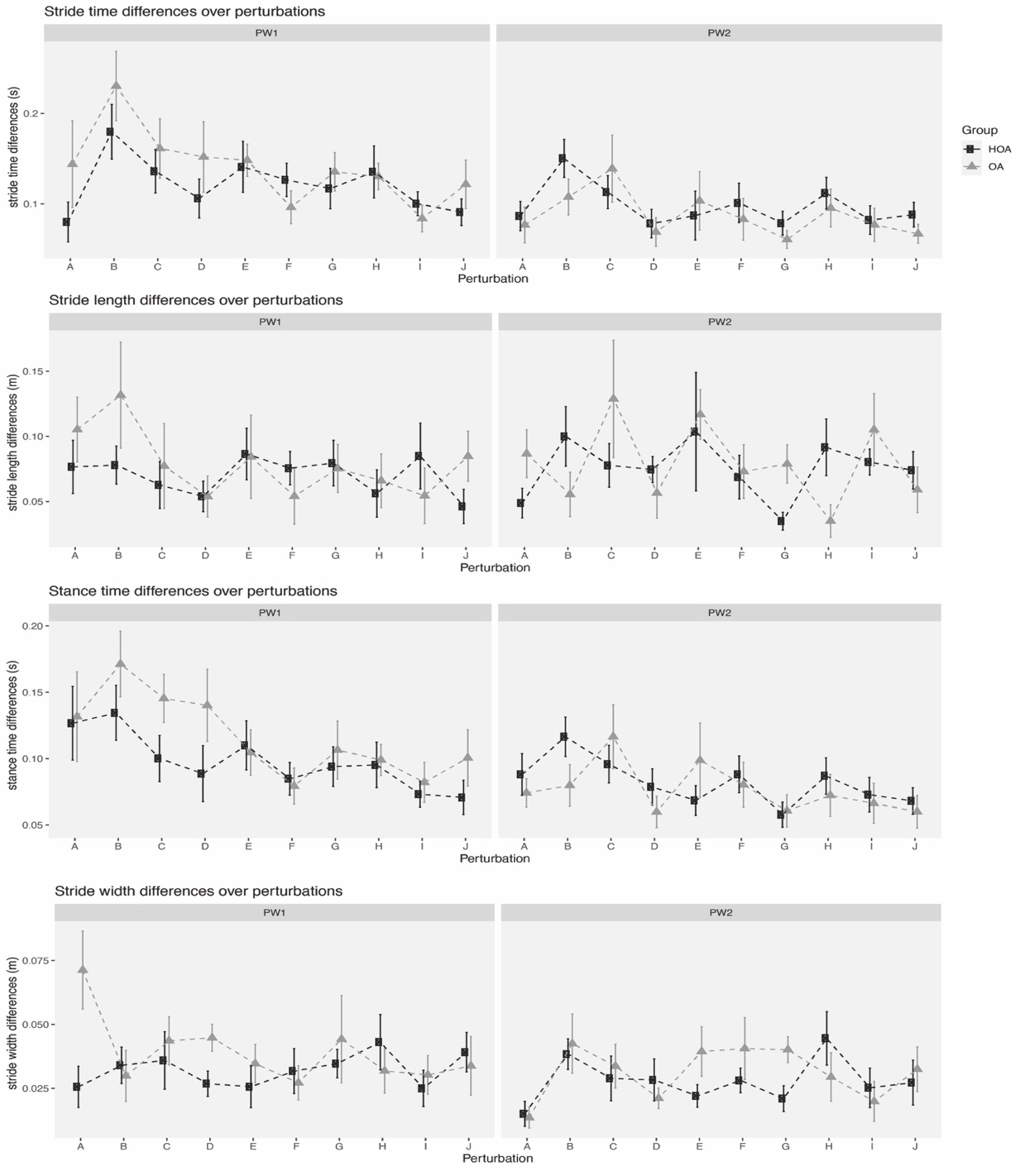


1. **Spearman Correlation results with linear fit, rho and p values:**
2. Non-significant negative association between mean deoxyhemoglobin and TUG-DT during CW2 in older women with and without OA after removing outliers:

rho= -0.39, p = 0.148

1. Significant negative association between mean oxyhemoglobin and BMI during PW2 in older women with and without OA:

rho= -0.66, p = 0.003

rho= -0.49, p = 0.04

1. Significant negative association between mean oxyhemoglobin and mean stance time during PW2 in older women with and without OA:

rho= -0.63, p = 0.008

1. **Table A showing description of physical and cognitive measures used in the study:**

| **Cognitive Assessment Measures** |  |
| --- | --- |
| Repeated Battery for the Assessment of Neuropsychological Status (RBANS)^1^ | The RBANS was designed to serve two main purposes: identifying and characterizing abnormal cognitive decline in older adults and providing a neuropsychological screening tool for younger individuals. The battery takes less than 30 minutes to complete and generates scaled scores across five cognitive domains: immediate memory, visuospatial/constructional abilities, language, attention, and delayed memory. The total RBANS score ranges from 40 to 160, with lower scores indicating greater levels of cognitive impairment. |
| Trail Making Test (TMT)^2^ | The TMT consists of two parts, and the time taken to complete each part assesses central executive functioning. In Part A (TMT-A), participants draw lines to connect consecutive numbers from 1 to 25. In Part B (TMT-B), participants connect numbers and letters in an alternating sequence: 1 to A, A to 2, 2 to B, and so forth. An average completion time for TMT-A is 29 seconds, with times exceeding 78 seconds considered deficient. For TMT-B, an average score is 75 seconds, while times over 273 seconds are deemed deficient. |
| **Physical Assessment Measures** |  |
| Mini- Balance Evaluation Systems Test (Mini-BEST)^3^ | The Mini-BEST assesses dynamic balance, functional mobility, and gait. It comprises 14 items divided into four sections: anticipatory, reactive postural control, sensory orientation, and dynamic gait. The total score for the Mini-BEST ranges from 0 to 28 points. |
| Short Physical Performance Battery Test (SPPB)^4^ | The SPPB test encompasses a series of measures that include gait speed, chair stand, and balance tests. It serves as a predictive tool for potential disability and helps monitor functional status in older adults. A score below 10 suggests the presence of one or more mobility limitations. |

**References:**

1. Randolph C, Tierney MC, Mohr E, Chase TN. The Repeatable Battery for the Assessment of Neuropsychological Status (RBANS): Preliminary Clinical Validity. *J Clin Exp Neuropsychol*. 1998;20(3):310-319. doi:10.1076/jcen.20.3.310.823

2. ASHENDORF L, JEFFERSON A, OCONNOR M, CHAISSON C, GREEN R, STERN R. Trail Making Test errors in normal aging, mild cognitive impairment, and dementia. *Archives of Clinical Neuropsychology*. Published online February 21, 2008. doi:10.1016/j.acn.2007.11.005

3. Yingyongyudha A, Saengsirisuwan V, Panichaporn W, Boonsinsukh R. The Mini-Balance Evaluation Systems Test (Mini-BESTest) Demonstrates Higher Accuracy in Identifying Older Adult Participants With History of Falls Than Do the BESTest, Berg Balance Scale, or Timed Up and Go Test. *Journal of Geriatric Physical Therapy*. 2016;39(2):64-70. doi:10.1519/JPT.0000000000000050

4. Veronese N, Bolzetta F, Toffanello ED, et al. Association Between Short Physical Performance Battery and Falls in Older People: The Progetto Veneto Anziani Study. *Rejuvenation Res*. 2014;17(3):276-284. doi:10.1089/rej.2013.1491
